# Antibiotic use in infants at risk of early-onset sepsis: results from a unicentric retrospective cohort study

**DOI:** 10.1186/s12887-024-04637-x

**Published:** 2024-04-05

**Authors:** Catalina Morales-Betancourt, Diego Fontiveros-Escalona, Adriana Montealegre-Pomar, Tania Carbayo-Jiménez, María Palomares-Eraso, Concepción de Alba-Romero, Elena Bergón-Sendín, Maria Teresa Moral Pumarega, Carmen Pallás-Alonso

**Affiliations:** 1 Department of Neonatology, 12 de Octubre University Hospital, Avenida de Córdoba S/N, Madrid, 28041 Spain; 2 Escuela de Doctorado, Universidad de Alcalá, Ciencias de la salud, Madrid, Spain; 3 San Ignacio University Hospital, Bogotá, Colombia; 4 Pontificia Universidad Javeriana, Bogotá, Colombia; 5 Primary Care Interventions to Prevent Maternal and Child Chronic Diseases of Perinatal and Developmental Origin (RICORS), Instituto de Salud Carlos III, RD21/0012/0009 Madrid, Spain

**Keywords:** Antibiotic use, Early-onset sepsis, Newborn, Antibiotics stewardship, Term infants

## Abstract

**Background:**

Antibiotic use for early-onset sepsis represents a high percentage of antibiotic consumption in the neonatal setting. Measures to assess infants at risk of early-onset sepsis are needed to optimize antibiotic use. Our primary objective was to assess the impact of a departmental guideline on antibiotic use among term infants with suspected EOS not confirmed, in our neonatal unit.

**Methods:**

Retrospective cohort study, to compare antibiotic use in term infants during a baseline period of January to December 2018, and a postintervention period from October 2019, to September 2020, respectively. The primary outcome was antibiotic use measured by days of therapy, the antibiotic spectrum index, the antibiotic use rate, and the length of therapy.

**Results:**

We included 71 infants in the baseline period and 66 infants in the postintervention period. Compared to those in the baseline period, there was a significant reduction in overall antibiotic measures in the postintervention period, (*P* < 0.001). The total days of therapy/1000 patient-days decreased from 63/1000 patient-days during the baseline period to 25.8/1000 patient-days in the postintervention period, representing a relative reduction of 59%. The antibiotic use rate decreased by more than half of the infants, from 3.2% during the baseline period to 1.3% in the postintervention period.

**Conclusions:**

The use of a departmental guideline to assess infants at risk of early-onset sepsis based on their clinical condition and prompt discontinuation of antibiotics, is a simple and low-cost measure that contributed to an important decrease in antibiotic use.

**Supplementary Information:**

The online version contains supplementary material available at 10.1186/s12887-024-04637-x.

## Background

The incidence of early-onset sepsis (EOS) has decreased in recent decades, and in 2018, the incidence reported in Spain was 1–1.2 per 1000 live births [1]. However, the incidence of suspected EOS is 6–16 times greater and represents approximately 10% of antibiotic use in the neonatal intensive care unit (NICU) [2].

The European Standards of Care for Newborn Health (ESCNH) highlights the role of reducing unnecessary or prolonged antibiotic therapy as a main step in improving health outcomes and decreasing the emergence of multidrug-resistant bacteria [3]. Multiple quality improvement initiatives and antibiotic stewardship programs have been trialed in order to reduce antibiotic overuse, however, most of them are based on a multivariate risk assessment using a risk calculator model [4, 5]. Despite the urge to optimize antibiotic consumption, the use of antibiotics in infants without proven EOS ranges from 70%, according to recent reports [6].

Our center has successfully implemented a quality improvement initiative to optimize antibiotic use in very low birth weight infants, through departmental guidelines and an affordable surveillance system, a significant reduction in antibiotic exposure was observed [7]. Following current evidence and previous experience, our NICU developed a quality improvement initiative to endorse the ESCNH standard for managing infants at risk of EOS through departmental guideline based on clinical observation and prompt antibiotic discontinuation.

The main objective of this study was to assess the impact of a departmental guideline on antibiotic use among term infants with suspected EOS not confirmed, in our NICU.

## Methods

### Setting and study design

This study was performed in the neonatology department of the 12 de Octubre University Hospital in Madrid, Spain. Our NICU is a IIIC-level unit. In our unit, the clinical observation of an infant with perinatal risk factors for EOS but a good appearance is routinely made while rooming-in with the mother. A medical examination is performed at 2 h after birth; if there are no signs of EOS, the mother–infant dyad is observed in the maternity ward until discharge.

We performed a retrospective cohort study to compare antibiotic use among term infants born between a baseline period of January to December 2018, and a postintervention period from October 2019, to September 2020.

We included all term newborn infants born in the maternity ward who had received antibiotics for suspected EOS during the first 3 days of life. We excluded outborn infants, those with major congenital malformations, and those treated for localized infections or surgical prophylaxis. We excluded infants with confirmed EOS from the analysis because antibiotic use is justified and the opportunity to reduce antibiotic overuse is limited.

### Implementation of a departmental guideline

Until 2019, the neonatology department didn’t have a guideline for the assessment of infants with risk factors for EOS and clinical signs. If an infant developed clinical signs or symptoms of EOS during admission, laboratory tests were performed, including blood cultures if antibiotics were to be started. The empiric antibiotic regimen for EOS included ampicillin, gentamycin. Cefotaxime was reserved for critically ill infants or those with suspected meningitis. If the blood culture was negative, antibiotic therapy was continued for 5–7 days.

In April 2019 a departmental guideline was developed for the assessment and clinical management of newborns born at > 35 weeks gestation who were at risk of EOS in line with the ESCNH (see flowchart in Additional file 1). The document defined standard antibiotic therapy durations and highlighted three key factors in the assessment of the symptomatic infants:

- Some signs and symptoms may be attributable to noninfectious conditions [8].
- The C-reactive protein (CRP) level may increase as an inflammatory marker; thus, the decision to start antibiotics should not rely only on CRP values [9].
- Antibiotics should be discontinued if blood cultures are negative at 36–48 h, and the use of third-generation cephalosporins should be avoided [9].

To facilitate implementation and uptake, we decided to allow a period of six months to pass before data collection.

### Measures

Our primary outcome was antibiotic use, which was assessed using the following measures: days of therapy (DOTs), DOTs/1000 patient-days (PDs), length of therapy (LOT), antibiotic use rate (AUR), antibiotic spectrum index (ASI) and ASI/LOT (see measures in Additional file 2) [10, 11].

As a secondary outcome, we analyzed the changes in hospital length of stay (LOS) as an indicator of the risk of unsafe care. In addition, we reviewed cases of missed EOS, which were identified by reviewing the clinical records through 28 days of life of all infants included in the study.

### Data collection and definitions

We considered the following risk factors for EOS: premature rupture of the membranes (ROM); a duration of ROM > 18 h before delivery; maternal *Streptococcus* group B (GBS) colonization with inadequate intrapartum antibiotic therapy; a history of previous GBS disease in offspring; and suspicion of intra-amniotic infection, which was defined by either a single maternal intrapartum temperature ≥39ºC or a temperature of 38–38.9 ºC for ≥ 30 min [12].

We defined suspected EOS as an evaluation for sepsis performed within 3 days of birth at the discretion of the attending neonatologist with empiric antibiotics. EOS was defined as sepsis diagnosed by blood culture within three days of birth [9].

### Statistical analyses

The descriptive statistics included the mean and standard deviation for normally distributed continuous variables, the median and interquartile range (IQR) for nonnormally distributed continuous variables, and the absolute and relative frequencies for categorical variables. Differences between groups were analyzed using the Mann–Whitney *U* test for quantitative variables and Fisher’s exact test or chi-squared test for categorical variables. The rate ratio and 95% confidence intervals were used to compare the DOTs and AUR between the baseline and postintervention periods. Statistical analyses were performed using Stata version 14 (StataCorp, LLC, College Station, TX).

## Results

### Patient characteristics

We identified 71 infants in the baseline period and 66 in the postintervention period. The perinatal and clinical characteristics of the included patients are summarized in Table 1.

**Table 1:** Demographics and characteristics of infants in the baseline and postintervention groups

	Study period			
Characteristic	**Baseline** *n* = 71	**Postintervention** *n* = 66	***p*** **value**	
Birthweight, g, mean (SD)	3494 (489)	3369 (451)	0.23	
Gestational age, weeks, mean (SD)	39.5 (1.1)	39.7 (1.3)	0.14	
Cesarean delivery	25 (35.2%)	26 (39.4%)	0.34	
Apgar, 5 min				
0–3	0 (0%)	2 (3%)	0.01	
4–6	6 (8.5%)	15 (22.7%)		
7–10	65 (91.6%)	49 (74.2%)		
GBS status				
Positive	15 (21%)	18 (27.3%)	0.57	
Negative	50 (70.4%)	45 (68.2%)		
Unknown	6 (8.5%)	3 (4.5%)		
Maternal temperature > 38ºC	30 (42.3%)	28 (42.4%)	0.98	
ROM > 18 h	21 (29.6%)	13 (19.7%)	0.18	
Intrapartum antibiotic therapy	38 (53.5%)	45 (68.2%)	0.08	
CRP, mg/dL				
Onset, median (IQR)	0.6 (0.1–2.2)	1.41 (0.04–3.60)	0.39	
Highest, median (IQR)	3.7 (2.9–6.7)	3.5 (1.8–5.7)	0.09	

SD, standard deviation; IQR, interquartile range; GBS, group B *Streptococcus*; ROM, rupture of membranes; CRP, C-reactive protein.

### Antibiotic use

The measures of antibiotic use are shown in Table 2. In the baseline period, there were 14,089 days of total LOS and 461 days of total LOT. In the post-intervention period, there were 12,880 days of total LOS and 173 days of total LOT.

**Table 2:** Antibiotic use in the baseline and postintervention periods

Measures	Baseline *n* = 71	Postintervention *n* = 66	Incidence rate difference 95% CI	Relative difference %	*p* value	
DOTs total	887.3	333				
DOT total/1000 PDs	63	25.8	37.1 (32.1–42)	59.1	< 0.001	
DOTs ampicillin DOTs ampicillin/1000 PDs	432.3 30.68	157.1 12.18	18.5 (15–22)	60.3	< 0.001	
DOTs gentamicin DOTs gentamicin/1000 PDs	439 31.1	168 13	18.1 (14.5–21.6)	58.1	< 0.001	
DOTs cephotaxime DOTs cephotaxime/1000 PDs	16 1.1	7.8 0.6	0.5 (0.19–1.2)	45.4	0.08	
AUR (%)	3.2	1.3	1.9 (1.6–2.3)	59.3	< 0.001	
ASI/LOT, median (IQR)	7.0 (6.3–7.0)	7.0 (6.7–7.0)	-		0.87	
LOT, days, median (IQR)	7 (6–7)	2.5 (2.0–3.0)	-		< 0.001	

DOTs, days of therapy; PDs, patient-days; AUR, antibiotic use rate; ASI, antibiotic spectrum index; LOT, length of therapy; IQR, interquartile range.

### Safety

The median LOS significantly decreased from 7 days (IQR, 7–8 days) in the baseline period to 5 days (IQR, 3–7 days) in the postintervention period (*p* < 0.001). None of the patients was readmitted within 28 days.

The incidence of EOS remained stable, and the cases were identified using the approach used in each period. In the baseline period there were 5 cases of EOS and 1 case in the postintervention period.

## Discussion

In this study, following the implementation of a new departmental guideline in line with the ESCNH standards, we observed a significant reduction in overall measures of antibiotic use. In the postintervention period, the use of antibiotics for EOS was halved without any increase in the LOS and there were no cases of missed EOS.

Current recommendations across different societies about managing suspected or proven EOS are homogeneous and concur about decreasing antibiotics overuse. In high-income countries with high rates of pregnancy follow-up and declining incidence rates of EOS, the approach to managing infants at risk should be oriented toward clinical observation, and early discontinuation of antibiotics [3].

One of the most studied strategies to decrease antibiotic use in the EOS framework involve the implementation of a multivariate risk assessment through a risk calculator. Although it is a tool that requires training and proper interpretation, the results are promising and have led many units to reduce antibiotic consumption [5, 13]. What seems to be a constant in the success of the approaches implemented in other units is the educational factor, by highlighting the importance of antibiotic stewardship and making the staph aware of the actions that needs to be taken. In our unit, the implementation of a simple departmental guideline contributed to a reduction in early antibiotic exposure, as indicated by a decreased DOT and less LOS.

A recent study of a large international network revealed that even in the infants treated with antibiotics but without EOS, the median LOT was 4 days, which diverges with the current recommendation for stopping antibiotics within 36–48 h [6, 9]. In our cohort the LOT in the post invention period significantly decreased to 2.5 days, which is lower that the median described by Giannoni et al. Additionally in that large cohort, for each infant with proven EOS, 58 infants received antibiotics, in contrast and in a smaller dataset, for each infant with proven EOS, we initiated treatment in 20 infants considering both periods. We also decreased our AUR to 1.3%, which is closer to the goal suggested by the same authors.

Although our sample size was limited, the LOS did not increase as an adverse event and there were no cases of readmission. Furthermore, our results are consistent with those obtained by other groups. As in our study, the implementation of a common guideline based on active antibiotic discontinuation in three Norwegian NICUs, was associated with a significant reduction in antibiotic therapy duration and a 37% reduction in DOT [4]. Likewise, a strategy based on serial physical examination for suspected EOS in term neonates, within a quality improvement initiative in a NICU in Stavanger, improved antibiotic use measures, reducing by half the antibiotic exposure in the first 3 days of life, declining from 2.9 to 1.3% [14].

Recently Stocker et al. addressed the basics of the decision-making process regarding early antibiotic use [15]. Based on our experience, we agree that a straightforward approach that includes an understanding of the unit’s baseline situation and staff training on initiation and early discontinuation of antibiotics may be the most effective way for NICUs to improve their antibiotic use indicators and, consequently, their results.

## Conclusions

With this initiative, we propose an approach for the management of infants at risk of EOS oriented toward clinical observation and prompt discontinuation of antibiotics. This affordable and simple approach based on an observation strategy has contributed to reduce unnecessary use of antibiotics in term infants, in line with the recommendations of the ESCNH. Although we look forward toward an ideal strategy for assessing these infants, one model will not fit all the neonatal units. As each unit individualizes and develops its own approach, based on the neonatal population and available resources, we will be closer to narrowing the population exposed to unnecessary antibiotics during the first days of life.

### Electronic supplementary material

Below is the link to the electronic supplementary material.

Supplementary Material 1

Supplementary Material 2

## Data Availability

All data analyzed during this study are included in this article. Further enquiries are available upon reasonable request to the correspondence author.

## Declarations

### Authors’ information

C.P.A, M.T.M.P and E.B.S. are associated with the network RICORS funded by the Recovery, Transformation, and Resilience Plan 2017–2020, ISCIII, and the European Union–Next Generation EU (ref. RD21/0012/0009). C.M.B. is a Ph.D. student of the Program of Health Sciences, Escuela de Doctorado, Universidad de Alcalá, Alcalá de Henares, Spain.

## Abbreviations

ASI: Antibiotic spectrum index
AUR: Antibiotic use rate
CRP: C-reactive protein
DOTs: Days of therapy
EOS: Early-onset sepsis
ESCHN: European Standards of Care for Newborn Health
GBS: Group B *Streptococcus*
IQR: Interquartile range
LOS: Length of stay
LOT: Length of therapy
NICU: Neonatal intensive care unit
PDs: Patient-days
ROM: Premature rupture of the membranes
