# Supplementary material for: Antibiotic use in infants at risk of early-onset sepsis: results from a unicentric retrospective cohort study
Source: BMC Pediatr. 2024 Apr 5;24:245. doi: 10.1186/s12887-024-04637-x (PMC10996240; doi:10.1186/s12887-024-04637-x)

**Additional file 1. Flowchart of departmental guideline for assessment and clinical management of newborns at >35 weeks’ gestation at risk of EOS.**


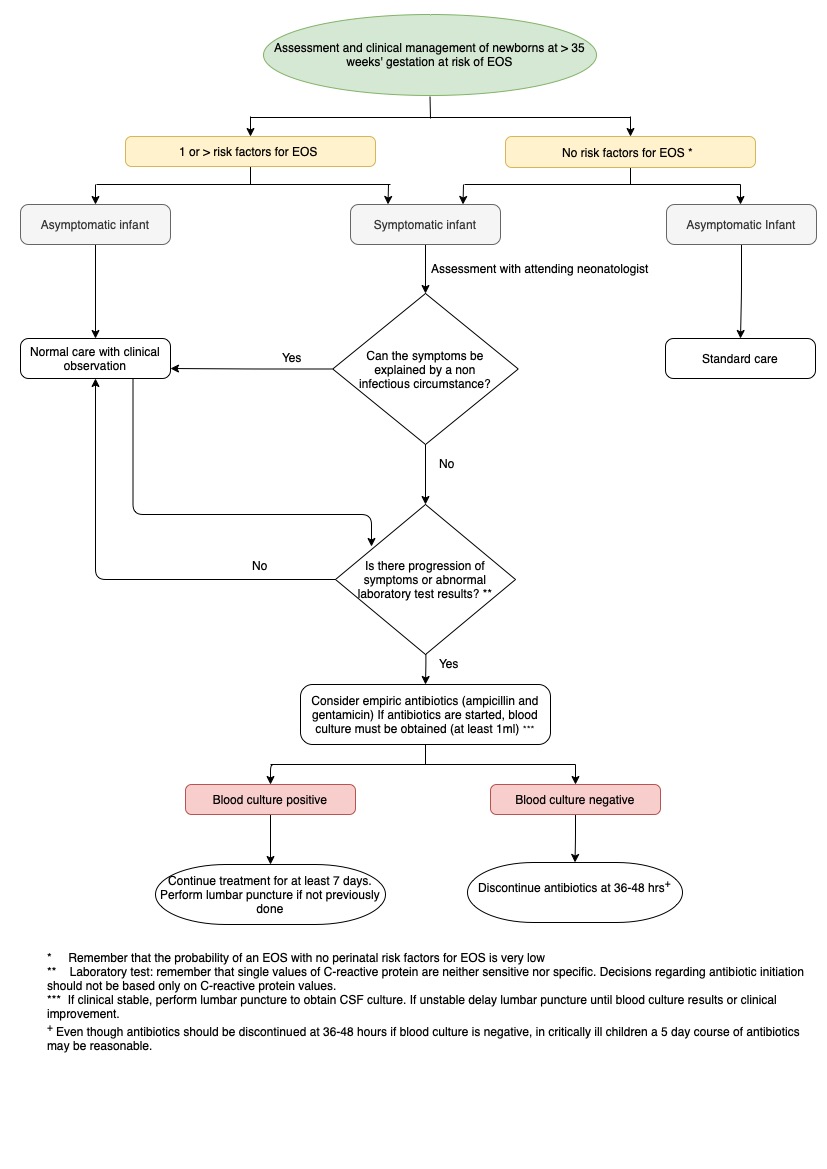

Supplement: Supplementary file 1 — Supplementary Material 1 [file 12887_2024_4637_MOESM1_ESM.docx]
