# Supplementary material for: Antibiotic use in infants at risk of early-onset sepsis: results from a unicentric retrospective cohort study
Source: BMC Pediatr. 2024 Apr 5;24:245. doi: 10.1186/s12887-024-04637-x (PMC10996240; doi:10.1186/s12887-024-04637-x)
Supplement: Supplementary file 2 — Supplementary Material 2 [file 12887_2024_4637_MOESM2_ESM.docx]

**Additional file 2. Antibiotic measures**

- Days of therapy (DOTs): is an aggregate sum of the days of exposure that accounts for each antibiotic and is calculated by multiplying the number of antibiotic doses by the dosing interval and dividing by 24 h. To calculate DOTs/1000 patient-days (PDs), PD is the product of the number of admitted patients and the mean length of stay, normalized to 1000 patients. This value was calculated individually and for all antibiotics.
- The length of therapy (LOT) was the number of calendar days that an infant received ≥1 antibiotics.
- The Antibiotic Spectrum Index (ASI): per antibiotic therapy day was calculated by multiplying the DOTs of each antibiotic by a score of 1–13 points, with a higher score signifying greater spectrum activity (ampicillin = 2 points, gentamicin = 5 points, cefotaxime = 5 points). The sum of the ASI per antibiotic received by the infant was then divided by the number of calendar days they received ≥1 antibiotics (ASI/LOT).
- The antibiotics use rate (AUR) was calculated by the sum of the number of days with ≥1 antibiotics used divided by the total LOS of all the infants admitted during each period and multiplied by 100 to be displayed as a percentage.
